# Characterizing microsatellite polymorphisms using assembly-based and mapping-based tools

**DOI:** 10.3906/biy-1903-16

**Published:** 2019-08-05

**Authors:** Gülfem DEMİR, Can ALKAN

**Affiliations:** 1 Department of Computer Engineering, Faculty of Engineering, Bilkent University, Bilkent, Ankara Turkey

**Keywords:** Microsatellites, genomics, whole genome sequencing

## Abstract

Microsatellite polymorphism has always been a challenge for genome assembly and sequence alignment due to sequencing errors, short read lengths, and high incidence of polymerase slippage in microsatellite regions. Despite the information they carry being very valuable, microsatellite variations have not gained enough attention to be a routine step in genome sequence analysis pipelines. After the completion of the 1000 Genomes Project, which aimed to establish the most detailed genetic variation catalog for humans, the consortium released only two microsatellite prediction sets generated by two tools. Many other large research efforts have failed to shed light on microsatellite variations. We evaluated the performance of three different local assembly methods on three different experimental settings, focusing on genotype-based performance, coverage impact, and preprocessing including flanking regions. All these experiments supported our initial expectations on assembly. We also demonstrate that overlap-layout-consensus (OLC)-basedassembly methods show higher sensitivity to microsatellite variant calling when compared to a de Bruijn graph-based approach. We conclude that assembly with OLC is the better method for genotyping microsatellites. Our pipeline is available at https://github.com/gulfemd/STRAssembly.

## 1. Introduction

One of the primary aims of genomics studies is to characterize genetic variations and associate them with phenotypes including genetic diseases. Recently there has been substantial progress in detecting various types of genetic variations (Shendure et al., 2017). Genome-wide association analyses have already identified thousands of genetic loci linked with human phenotypes, diseases, complex traits, and disorders. While many different types of genetic variations such as single-nucleotide polymorphisms (SNPs), copy number variation (CNV), and structural variation (SV) have been identified by these studies, microsatellite polymorphism remains largely understudied (Gymrek et al., 2016). For example, The 1000 Genomes Project (The 1000 Genomes Project Consortium, 2015), which aimed to establish the most detailed genetic variation catalog for humans, analyzed 2504 individuals from 26 populations and only reported SNPs, indels, and a limited number of types of structural variation (i.e. deletions, small inversions, mobile element insertions, and tandem duplications) in detail. The consortium has released only two microsatellite polymorphism call sets, identified using two algorithms, namely lobSTR (Gymrek et al., 2012) and RepeatSeq (Highnam et al., 2013). The 1000 Genomes Project and other large research efforts had limited effect on shedding light on microsatellite polymorphism.The major obstacle in this endeavor is the complex nature of microsatellites. Being a rich primary source of genetic variation, single nucleotide changes are probably the simplest type and easiest to assay. On the other hand, microsatellites are composed of a few nucleotides that are repeated several times. This structure causes a high mutation rate, which can reach 1/500 mutations per locus per generation. This is 200× higher than the rate of CNVs and 200,000× higher than the rate of de novo single-nucleotide variants. Their hypervariability and ubiquity throughout the genome makes them difficult to characterize. Despite being harder to identify, microsatellites are still highly utilized in human genetics applications, including forensics (Gill, 2002) and medical genetics (Willems et al., 2014), since they serve as a major source of genetic polymorphism among individuals, as detailed below:● Forensics: Microsatellite analysis is the de facto standard for constructing national public forensic DNA databases (Gill, 2002). Microsatellites usually have a small number of alleles, which increase the information entropy of a single microsatellite region. This means that a limited number of microsatellites can sufficiently identify a single individual. During the late 1990s, the FBI Laboratory established the CODIS set. Despite only containing 13 microsatellite loci, the CODIS set is recognized as the standard for human identification.● Medical genetics: Microsatellite mutations have been associated with more than 40 single-gene disorders (Willems et al., 2014), such as Huntington’s disease and amyotrophic lateral sclerosis/frontotemporal dementia (ALS/FTD). In the case of ALS, the condition is triggered by the abnormal expansion of short repeat units (Doi et al., 2014). In addition to single-gene disorders, microsatellites also contribute to the heritability of various complex traits. 

### 1.1. Motivation

Microsatellite polymorphisms are associated with several genetic disorders. Among those, dentatorubral-pallidoluysian atrophy (DRPLA) is a rare brain disorder that mainly impacts the mental and emotional state and intellectual ability in the patient, and causes uncontrollable muscle movements. It is associated with the expansion of the CAG**microsatellite (over 49-88 copies) in the atrophin 1 (*ATN1*) gene (Mongelli et al., 2018). Similarly, the mutated androgen receptor (*AR*) gene with an expanded CAG**microsatellite (40 to 62 copies) in the coding region is shown to be responsible for the pathogenesis of spinal-bulbar muscular atrophy (SBMA), in which loss of motor neurons affects the voluntary muscle movement in the face, mouth, and throat (Kozlowski et al., 2010). Therefore, it is of clinical importance to accurately and quickly analyze microsatellite polymorphisms.There are several other genetic diseases linked with microsatellite polymorphisms that cover ≈3% of the sequenced human genome, making microsatellite detection research even more significant (Usdin, 2008). These patterns are also a major cause of ambiguity in genome assembly and sequence alignment, which may cause inaccurate interpretations. Hence, microsatellite polymorphisms, due to their repetitive nature, have always been a challenge for genome assembly and sequence alignment (Treangen and Salzberg, 2012). Because of this, microsatellite polymorphisms are relatively unexplored and are lacking in large-scale analyses, when other types of variations (e.g., SNPs, CNVs, insertions, and deletions) have been comprehensively cataloged in extensive studies (Willems et al., 2014; The 1000 Genomes Project Consortium, 2015).

### 1.2. Identification of microsatellite polymorphisms

Microsatellites in the genome of an organism may be identified using two different approaches: (i) analyzing de novo assemblies, and (ii) using resequencing data and a reference genome.The first approach starts by building a de novo assembly of the genome to be analyzed. To achieve this, any genome assembly algorithm can be used (see Section 1.3 below for a discussion on assembly algorithms). After the reads are assembled into longer contiguous DNA segments called contigs, the microsatellites can be identified using a tandem repeat discovery algorithm. The most commonly used algorithm for this purpose is Tandem Repeats Finder (Benson, 1999), which is still employed for new versions of the human genome. Tandem Repeats Finder is a greedy algorithm that scans the genome using different window sizes and tries to find whether two or more adjacent windows contain highly similar sequences. Other tools that can be applied include REPuter (Kurtz et al., 2001) and Look4TRs (Velasco et al., 2018). REPuter also uses a greedy strategy: it first finds maximal exact repeats, and then tries to extend the repeats to include mismatch and indels. On the other hand, Look4TRs is a more involved algorithm and it uses self-supervised hidden Markov models to find microsatellites.The first approach we outlined above relies on highly accurate assemblies, which makes it useful for newly constructed high-quality reference genomes. However, when there is already a reference genome available, such as the human genome, constructing de novo assemblies for additional individuals is both costly and generates fragmented low-quality assemblies due to repeats (Treangen and Salzberg, 2012).Because of the problems of accurate de novo assembly construction, the second approach is used when analyzing microsatellite polymorphisms. This approach involves first aligning the reads to a reference genome using a read aligner such as BWA, and then searching for inconsistencies between the read and the aligned portion of the reference. We provide more details about this approach below.Although generic indel calling tools can be used to detect microsatellite polymorphisms, they do not perform as well as specialized tools such as lobSTR (Gymrek et al., 2012) and RepeatSeq (Highnam et al., 2013), both of which are microsatellite polymorphism callers using high-throughput sequencing (HTS) data and split-read signature (Alkan et al., 2011). However, there are only a limited number of tools available that have been developed specifically for detecting microsatellite polymorphisms and to the best of our knowledge, none of them utilize local genome assembly methods during variant calling phase (Cao et al., 2015). Most microsatellite polymorphism callers try to identify variation by comparing a read sequence with a reference sequence. Since they expect reads to be longer than the regions that encompass microsatellites, this approach significantly limits the detectable microsatellite length. Using local genome assembly information enables us to be able to identify longer microsatellite polymorphisms. There is still need for improvement in microsatellite characterization in newly sequenced individual genomes using HTS, especially identification of de novo expansions and contractions, which is crucial for many applications in biology, such as medical genetics, forensics, and population genetics. Here we aim to improve the accuracy of microsatellite copy number detection by using local genome assembly.

### 1.3. Assembly algorithms

There are many (>30) assembly tools that use different algorithms and data structures to optimize their resource requirements. A nonexhaustive chronological list of sequence assembly tools for large genomes using short reads includes ABySS (Simpson et al., 2009), SGA (Simpson and Durbin, 2012), SOAPdenovo (Luo et al., 2012), Minia (Chikhi and Rizk, 2013), DISCOVAR (Weisenfeld et al., 2014), and BCALM2 (Chikhi et al., 2016). ABySS was the first tool to assemble a whole human genome from short reads by distributing a de Bruijn graph across a cluster of nodes (Simpson et al., 2009). A more recent tool, Minia, also uses de Bruijn graphs but reduces memory requirements by using a Bloom filter (Bloom, 1970), which is a space-efficient hash-based data structure to test existence of an element in a set. Peak memory usage for Minia is 5.7 GB, whereas the memory consumption of ABySS goes up to 336 GB for de novo human genome assembly. However, this a trade-off; lower memory usage incurs run-time costs: execution times for ABySS and Minia are 23 h and 15 h, respectively. On the other hand, a recent study (Cherukuri and Janga, 2016) showed that overlap-layout-consensus (OLC)-based methods are able to assemble the human genome sequence with an order of magnitude better in terms of contiguity to the de Bruijn graph approach. Therefore, genome assembly accuracy depends on the strategy used to generate the assembly.

### 1.4. Challenge

Most microsatellite regions are difficult to characterize using short Illumina reads, which are generally up to 150 base pairs in length. Although sequencing technologies that produce longer reads, such as PacBio and Oxford Nanopore, are becoming popular, they still generate reads with high indel error rates at higher costs. Furthermore, if the microsatellite region is longer than the read length, aligners cannot map the reads uniquely. Another crucial challenge is that microsatellite sequencing data include polymerase chain reaction (PCR) stutter artifacts (Litt et al., 1993), which incorrectly generate reads that include incorrect copy numbers of microsatellites compared to the underlying DNA sequence. Although there has been considerable effort in understanding the nature of sequencing errors, variant calling pipelines still suffer from them.

### 1.5. Contributions

In this study we used local assembly methods to characterize microsatellites. To summarize:● We developed a pipeline using existing tools that starts from raw reads to genotype microsatellites. We integrated local assembly as a new step in this pipeline.● We demonstrated that using local sequence assembly on microsatellite regions may help variant callers increase sensitivity.● We evaluated assembly methods that make use of graph data structures, namely de Bruijn graph and OLC-based approaches.● We analyzed the significance of read coverage in microsatellite detection.

## 2. Materials and methods

**Figure 1:**
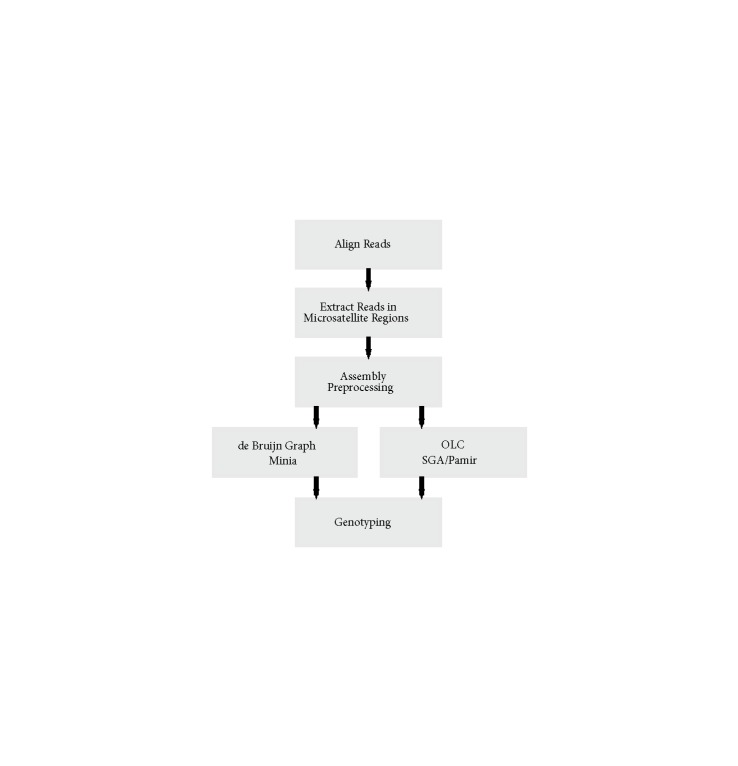
Microsatellite characterization pipeline using local assembly.

The main aim of this work is to use sequence assembly methods for regions that are known to harbor microsatellites based on the reference genome and build a complete pipeline that starts from the reads generated from a sample to genotype microsatellite polymorphisms. In light of the information about genome assembly tools outlined above, we have selected three different assemblers to be integrated into our pipeline: SGA (Simpson and Durbin, 2012) (an OLC-based de novo assembler), Minia (a de Bruijn based de novo assembler), and Pamir (Kavak et al., 2017) (an OLC-based local assembler). Here we aimed to include one tool from each possible assembly strategy (de Bruijn, OLC-de novo, and OLC-local). Pamir is the only OLC-based local assembly tool and SGA is the only OLC-based de novo assembler developed for Illumina. Although there are many de Bruijn graph-based assemblers, we selected Minia because of its low memory footprint. Briefly, our method is composed of the following steps (Figure  1):1. Align reads to the reference genome. 2. Extract reads that map to close proximity to, and within, known microsatellite regions.3. Preprocess reads before assembly.4. Assemble extracted sequences using SGA, Minia, or Pamir.5. Predict genotypes (i.e. heterozygous vs. homozygous).Below, we first describe how we generate the simulated datasets to test our methods, and then we give details on the microsatellite polymorphism characterization pipeline.

### 2.1. Simulations

To test our pipeline and the effect of different assemblers, we simulated microsatellite polymorphismsusing the human reference genome (GRCh38). In this manuscript we compare our local assembly-based pipeline using three different assemblers (Pamir, Minia, and SGA) with an alternative standalone microsatellite polymorphism assessment tool (lobSTR). Note that there are no gold standard truth sets for microsatellite polymorphisms generated from the genomes of biological samples; therefore, we opted for a simulation-based strategy to evaluate pipeline efficacy. We based our simulations on human chromosome 20 (NCBI Accession ID: CM000682.2) since it is the shortest chromosome in the current human genome reference assembly. We first downloaded microsatellite annotations for chromosome 20 of GRCh38 generated using Tandem Repeats Finder (TRF) (Benson, 1999). We then filtered for annotations where (i) repeat matches are perfect (i.e. there are no changes in repeat units), and (ii) both copy number and repeat unit lengths are greater than 3. We therefore obtained 1963 microsatellite regions for chromosome 20, which we then used for a random polymorphism simulation based on previously observed copy numbers of microsatellites from a genome-wide study (Willems et al., 2017). In order to test for reproducibility, we repeated the microsatellite annotation analysis using Look4TRs (Velasco et al., 2018) as an alternative tandem repeat finder. We used Look4TRs to characterize microsatellites within the same human chromosome 20 sequence, and after applying the same filters as described above, we obtained the same set of microsatellite regions that we generated using TRF. Therefore, our results are the same with this set of annotations.Using these regions, we simulated multiple versions of polymorphisms to generate synthetically expanded microsatellites. The simulation accepts microsatellite regions (TRF output) and the reference genome as input and produces two versions of the reference (i.e. corresponding to maternal and paternal DNA) together with metadata about expanded regions (e.g., coordinates, new copy number, and genotype). Our workflow in this step for each microsatellite region is as follows: (i) randomly choose between homozygous or heterozygous genotype, (ii) randomly pick an expansion factor N between 1 and 30, (iii) identify the microsatellite region in the reference genome, and (iv) inflate the sequence by inserting N more repeat units. If the genotype is simulated to be homozygous, both alleles have the same expansion (i.e. same sequence). If it is heterozygous, one allele might be the same as the reference genome while the other one has a random expansion, or they both can have different random expansions. It is common to use synthetic reads in testing bioinformatics pipelines. For this purpose, after simulating the polymorphisms, we generated short Illumina sequences using Mitty (https://github.com/sbg/Mitty). For all our simulation experiments, we used the built-in error model for the IlluminaHiSeq X platform and generated reads with varying depths of coverage. In this simulation, we set theread-length parameter to 150 bp and the average fragment size to 350 bp.

### 2.2. Microsatellite detection pipeline

#### 2.2.1. Read mapping

We first align the reads to the reference human genome using a standard read mapper, namely BWA-MEM (Li et al., 2014). Following the standard procedures of HTS read alignment (The 1000 Genomes Project Consortium, 2015), we convert the output to the BAM format and then sort and remove PCR duplicates using SAMtools (Li et al., 2009).

#### 2.2.2. Preparing data for assembly

In this step, we collect the reads needed in the assembly process. We first extract reads that map to the known microsatellite regions using the HTSlib library to process HTS data (http://www.htslib.org). For each such read, we also check whether the mapping supports a perfect match to the reference or shows a microsatellite polymorphism. We collect this information from the Concise Idiosyncratic Gapped Alignment Report (CIGAR) string as reported by BWA-MEM. Then, for each microsatellite region, if the region includes at least 50% of mapped reads that are the same as the reference, we conclude that this region has at least one reference allele and remove these reads from consideration to reduce the computational load. Finally, we output the reads and their map locations in FASTQ and SAM formats ready for the assembly step.

#### 2.2.3. Assembly

As we discussed above, we use SGA (Simpson and Durbin, 2012), Minia (Chikhi et al., 2016), and Pamir (Kavak et al., 2017) as alternative assemblers in this project. We assemble the FASTQ files generated in the previous step using each tool with default options.

#### 2.2.4. Genotyping

In this step we predict the genotype of the microsatellite polymorphism for the analyzed sample; i.e. we calculate whether the sample is homozygous for the reference allele, heterozygous, or homozygous for the alternative allele. Here we apply a simple calculation for the genotype support. Note that if we observe a sufficiently high number of reads supporting the reference allele in the preprocessing step, we mark this region to include at least one reference allele. For other cases, our genotyping method is as follows. In the case of homozygosity (either reference or alternative), the assembler in the previous step generates only one contiguous sequence (contig). Similarly, it generates two contigs in the case of heterozygosity. However, it is also possible for the assembler to report more contigs due to sequence errors and microsatellite sequence complexity. In such cases, we realign the reads to all contigs for the microsatellite region in question using BWA-MEM. We then select the two contigs with the highest amount of read support. If one of the contigs has very low support (<30 of the reads), we then predict the variation to be homozygous for the higher-support contig. Otherwise we report the variation to be heterozygous.

## 3. Results

We tested our pipeline using a simulated dataset (Section 2) and compared its performance with lobSTR (Gymrek et al., 2012). We performed three experiments for:1. Evaluating methods based on their performance on separate genotypes.2. Analyzing how sequence coverage impacts assembly-based callers.3. Assessing the importance of preprocessing and including flanking regions.

### 3.1. Genotyping performance

**Figure 2:**
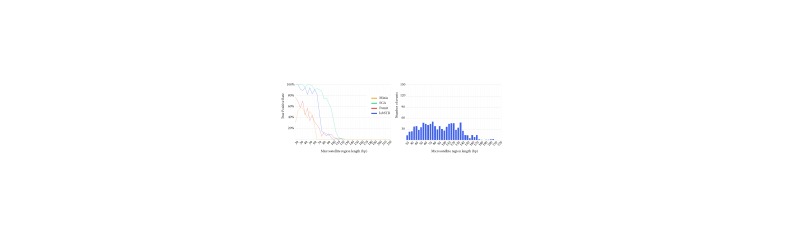
Results for homozygous events. True positive rates vs. region length (left), and number of homozygous events vs. region length (right).

**Figure 3:**
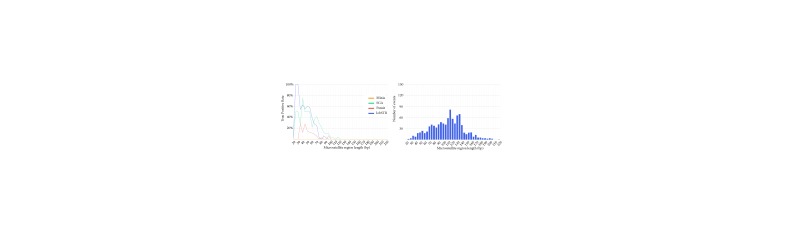
Results for heterozygous events. True positive rates vs. region length (left), and number of heterozygous events vs. region length (right).

**Figure 4:**
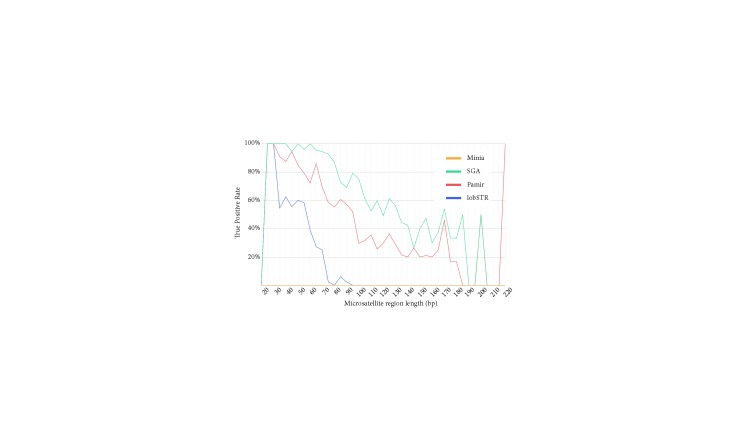
True positive rates for partially detected heterozygous events vs. region length.

**Figure 5:**
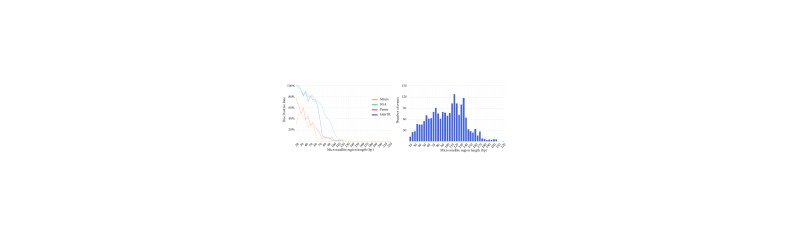
Results for all events. True positive rates vs. region length (left), and number of heterozygous events vs. region length (right).

In this experiment we used the simulated events based on 1963 microsatellite regions in GRCh38 (see Section 2), and reads simulated at a depth of coverage of 60×. Each region was inflated by a random amount of copy numbers (between 1 and 30) and assigned a random genotype (970 homozygous, 993 heterozygous). In all heterozygous events, alleles from each parent have different copy numbers and both of them are alternate (i.e. different from reference) alleles.We report true positive rates (TPRs) of our pipeline using each different assembler in addition to lobSTR as a distribution over microsatellite region size (i.e. copy number × microsatellite unit length) in Table 1. We also group the events into bins based on microsatellite region size. In the simulation experiments, the shortest and longest microsatellite regions were 20 bp and 220 bp, respectively. Briefly, our pipeline with SGA was the most successful at calling homozygous microsatellites, followed by lobSTR (Figure  2). Additionally, we observed that the SGA-based pipeline shows similar accuracy across the widest range of microsatellite region length.Figures 3 and 4 depict the true positive ratios of full and partial hit rates of heterozygous events.For a heterozygous microsatellite polymorphism, if a caller is able to determine the copy number for both alleles, we considered that prediction as a hit. On the other hand, if it calls only one of the alleles correctly, we consider it a partial hit. Once again, SGA has proven to be the most powerful in detecting at least one of the alleles correctly. However, especially in shorter microsatellite regions, lobSTR performed significantly better, but its accuracy is affected negatively in regions longer than 65 bp.In general, considering lobSTR’s 0.349 true positive rate in homozygous events versus 0.080 in heterozygous cases, it shows a substantial disadvantage in calling heterozygous microsatellite polymorphisms. Our pipeline with Minia and Pamir assemblers performed poorly in all cases (Figure  5) with similar true positive rates (0.066 and 0.108, respectively). Although all approaches have at least 4× lower TPR in heterozygous microsatellites compared to homozygous, Minia (a de Bruijn graph-based tool) failed to detect even one heterozygous variant.We have also examined each tool’s ability to accurately predict genotype (i.e. reporting as heterozygous or homozygous) of microsatellite regions without taking the reported copy numbers into account (Table 2). lobSTR reported only 76 of 993 heterozygous events correctly as heterozygous, while Pamir is the best-performing with 628 correct predictions. On the other hand, SGA is better at annotating homozygous regions. Based on these results, we conclude that assembly-based methods are superior to lobSTR, which shows that characterizing different alleles is more accurate with local assembly.

**Table 1:** **True positive rates for all events**

	Homozygous (n = 970)		Heterozygous (n = 993)	
Tool	Correct call	Correct call ratio	Correct call	Correct call ratio
Minia	474	49%	560	56%
SGA	725	75%	517	52%
Pamir	432	45%	628	63%
lobSTR	359	37%	76	8%

**Table 2:** Summary of genotype calls in all events.

	40×		60×		80×	
Tool	True	TPR	True	TPR	True	TPR
Minia	142	7.2%	194	9.9%	224	11.4%
SGA	568	29%	793	40.4%	892	45.5%
Pamir	92	4.7%	289	14.7%	331	16.9%
lobSTR	487	24.8%	589	30%	615	31.3%

### 3.2. Coverage tests

**Figure 6:**
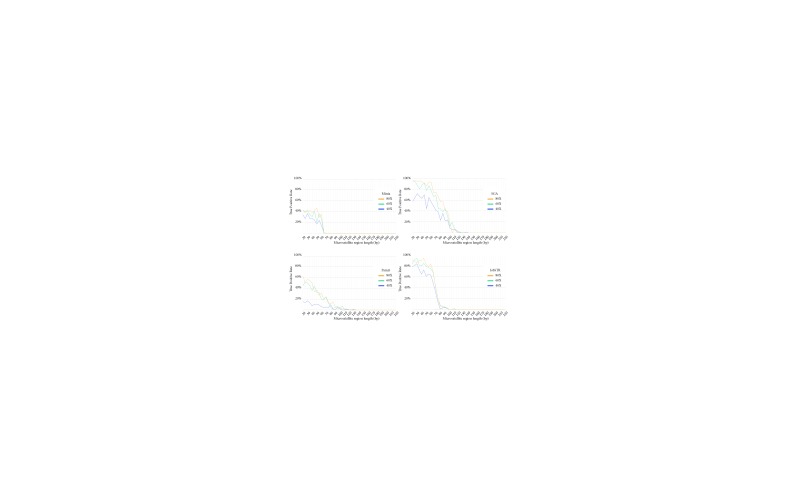
True positive rates of Minia, SGA, Pamir, and lobSTR with different depths of coverage binned in various microsatellite region lengths.

Next, we aimed to assess the impact of depth of coverage on the accuracy of microsatellite polymorphism detection. We simulated sequence data from the altered genome with various depths of coverage (40×, 60×, and 80×). True positive rates for each caller with different depths are shown in Figure  6.As expected, higher coverage helps improve the performance of all methods (Table 3). However, the gain in recall is different between 40× to 60× and 60× to 80×. This result suggests that improvement in prediction accuracy saturates at around 80× depth coverage. For example, SGA was able to call 1.39× more events with 60× coverage when compared to 40× coverage; however, the gain in TPR is only 1.12× when the coverage increases from 60× to 80×.We also observed that SGA and lobSTR demonstrated good recall rates at low coverage compared to others. Pamir’s recall rate tripled with 60× coverage, compared to 40×, where lobSTR results did not change drastically across different depths of coverage. Therefore, higher depth of coverage data is more important for assembly-based methods.De Bruijn graph-based Minia once again showed the poorest performance, and it could only characterize short (<70 bp) microsatellite regions. This is expected due to the assembly collisions in repetitive regions (Zerbino and Birney, 2008).

**Table 3:** **True positive rates for 40×, 60×, and 80× coverage.**

	Homozygous			Heterozygous					Total				
Tool	Hit	Sim.	Hit TPR	Hit	PHit	Sim.	Hit TPR	PHit TPR	Hit	PHit	Sim.	Hit TPR	PHit TPR
Minia	130	970	0.134	0	0	993	0	0	130	130	1,963	0.066	0.066
SGA	514	970	0.530	108	642	993	0.109	0.647	622	1,156	1,963	0.317	0.589
Pamir	187	970	0.193	25	419	993	0.025	0.422	212	606	1,963	0.108	0.309
lobSTR	339	970	0349	79	79	993	0.080	0.080	418	418	1,963	0.213	0.213

### 3.3. Effects of preprocessing

**Figure 7:**
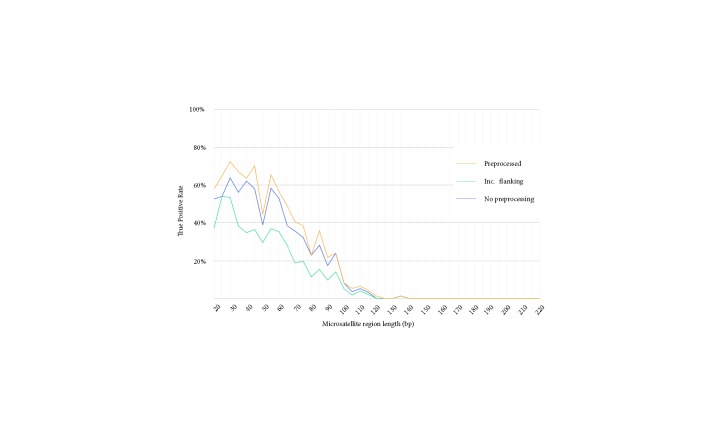
True positive rates of our pipeline using SGA with different setups binned in various microsatellite region lengths.

As the final experiment, we tested a single tool with different configurations. We selected SGA for this purpose as it showed the best performance in accuracy. Here we aimed to assess the possibility of tuning SGA to further improve its sensitivity. We used the same sets of data and microsatellite regions to test:● SGA pipeline with preprocessing, same as in previous experiments.● SGA pipeline without the preprocessing step.● SGA pipeline with both flanking regions of each microsatellite region.We report the true positive rates for these settings in Figure  7. We observe that including flanking regions in fact lowered the accuracy of microsatellite polymorphism characterization. This is probably due to increased sequence complexity in longer microsatellite regions. On the other hand, SGA showed the best performance with preprocessed assembly.

## 4. Discussion

In this paper we addressed the problem of characterizing microsatellites, important sources of genetic variation that are not fully addressed in large scale genome projects. To help improve microsatellite polymorphism discovery with short read data we proposed an end-to-end solution for using local assembly, and compared it against a mapping-based solution. We concluded that our proposed pipeline with the SGA (Simpson and Durbin, 2012) assembler produced better results than several other assembly tools and a state-of-the-art microsatellite caller in estimating copy numbers. However, since SGA uses a relatively computationally costly OLC approach, it is slower than the alternatives we analyzed in this study. On the other hand, lobSTR and Pamir run times are comparable, and they are also considerably faster than Minia. Given the lack of accurate microsatellite characterization, we believe that the correctness and comprehensiveness of detecting these variants is more valuable than time complexity.State-of-the-art microsatellite callers that do not make use of local assembly expect microsatellite regions to be covered entirely together with flanking regions. All of our experiments demonstrated that for reads of length 150 bp, the “discoverable” microsatellite region size is ≤70 using mapping based callers. We showed that OLC-based assembly methods benefit from longer reads, more so than mapping or de Bruijn graph-based approaches (Miller et al., 2010). We observed from depth of coverage tests that higher sequence coverage improves the sensitivity of all approaches. However, the gain in the number of microsatellite regions correctly predicted does not scale linearly, which indicates a potential upper bound of sensitivity for HTS analysis.There are two main directions that we can take to further improve assembly-based microsatellite calling pipelines. First, to lower the false negative rate, we can include one-end anchored reads (Kavak et al., 2017), which are defined as the paired-end reads where only one end can be mapped to the reference genome. In this study, we only used reads that map to a microsatellite region. Since most microsatellite regions are shorter than the fragment length, and the microsatellite regions are repetitive with high sequence identity, a case where both paired-ends do not map to the reference genome due to an expansion is unlikely. Hence, one-end anchored reads will be helpful in discovering microsatellite variations. Second, to achieve an accurate copy number estimation we can improve alignment quality of sequence reads. This not only applies to assembly-based pipelines but also applies to tools depending on the alignment step, such as lobSTR. A recent study proposes a dynamic programming-based algorithm for the realignment step, where repeat patterns in microsatellite regions are given as prior knowledge, and these patterns are used multiple times in the realignment process in order to achieve more accurate alignments of microsatellite-containing reads (Kojima et al., 2016). Since the assembly-based pipeline that we propose also uses alignments after contig generation, better realignment would help obtain more accurate calls.

## Acknowledgments

We thank Fereydoun Hormozdiari and Marzieh Eslami Rasekh for their support in analyzing data during the course of this project. This study was supported by the Scientific and Technological Research Council of Turkey (TÜBİTAK) via grant 215E172 to the second author.

## References

[ref1] (2011). Genome structural variation discovery and genotyping. Nature Review Genetics.

[ref2] (1999). Tandem repeats finder: a program to analyze DNA sequences. Nucleic Acids Research.

[ref3] (1970). Space/time trade-offs in hash coding with allowable errors. Communications of the ACM.

[ref4] (2015). Sequencing technologies and tools for short tandem repeat variation detection. Briefings in Bioinformatics.

[ref5] (2016). Benchmarking of de novo assembly algorithms for nanopore data reveals optimal performance of OLC approaches. BMC Genomics.

[ref6] (2016). Compacting de Bruijn graphs from sequencing data quickly and in low memory. Bioinformatics.

[ref7] (2013). Space-efficient and exact de Bruijn graph representation based on a Bloom filter. Algorithms for Molecular Biology.

[ref8] (2014). Rapid detection of expanded short tandem repeats in personal genomics using hybrid sequencing. Bioinformatics.

[ref9] (2002). Role of short tandem repeat DNA in forensic casework in the UK-past, present, and future perspectives. BioTechniques.

[ref10] (2012). lobSTR: A short tandem repeat profiler for personal genomes. Genome Research.

[ref11] (2016). Abundant contribution of short tandem repeats to gene expression variation in humans. Nature Genetics.

[ref12] (2013). Accurate human microsatellite genotypes from high-throughput resequencing data using informed error profiles. Nucleic Acids Research.

[ref13] (2017). Discovery and genotyping of novel sequence insertions in many sequenced individuals. Bioinformatics.

[ref14] (2016). STR-realigner: a realignment method for short tandem repeat regions. BMC Genomics.

[ref15] (2010). Trinucleotide repeats: triggers for genomic disorders. Genome Medicine.

[ref16] (2001). REPuter: the manifold applications of repeat analysis on a genomic scale. Nucleic Acids Research.

[ref17] (2014). Relationship estimation from whole-genome sequence data. PLoS Genetics.

[ref18] (2009). The sequence alignment/map format and SAMtools. Bioinformatics.

[ref19] (1993). Shadow bands seen when typing polymorphic dinucleotide repeats: some causes and cures. BioTechniques.

[ref20] (2012). SOAPdenovo2: an empirically improved memory-efficient short-read de novo assembler. GigaScience.

[ref21] (2010). Assembly algorithms for next-generation sequencing data. Genomics.

[ref22] (2018). Multiple system atrophy and CAG repeat length: A genetic screening of polyglutamine disease genes in Italian patients. Neuroscience Letters.

[ref23] (2017). DNA sequencing at 40: past, present and future. Nature.

[ref24] (2012). Efficient de novo assembly of large genomes using compressed data structures. Genome Research.

[ref25] (2009). AbySS: a parallel assembler for short read sequence data. Genome Research.

[ref26] (2015). A global reference for human genetic variation. Nature.

[ref27] (2012). Repetitive DNA and next-generation sequencing: computational challenges and solutions. Nature Reviews Genetics.

[ref28] (2008). The biological effects of simple tandem repeats: lessons from the repeat expansion diseases. Genome Research.

[ref29] (2018). Look4TRs: A de novo tool for detecting simple tandem repeats using self-supervised hidden Markov models.

[ref30] (2014). Comprehensive variation discovery in single human genomes. Nature Genetics.

[ref31] (2014). The 1000 Genomes Consortium. Genome Research.

[ref32] (2008). Velvet: algorithms for de novo short read assembly using de Bruijn graphs. Genome Research.

